# Effects of osmolality and solutes on the morphology of red blood cells according to three-dimensional refractive index tomography

**DOI:** 10.1371/journal.pone.0262106

**Published:** 2021-12-31

**Authors:** Minkook Son, Ye Sung Lee, Mahn Jae Lee, YongKeun Park, Hae-Rahn Bae, Seung Yeob Lee, Myung-Geun Shin, Sung Yang

**Affiliations:** 1 Department of Biomedical Science and Engineering, Gwangju Institute of Science and Technology, Gwangju, Republic of Korea; 2 School of Mechanical Engineering, Gwangju Institute of Science and Technology, Gwangju, Republic of Korea; 3 Graduate School of Medical Science and Engineering, Korea Advanced Institute of Science and Technology, Daejeon, Republic of Korea; 4 Department of Physics, Korea Advanced Institute of Science and Technology, Daejeon, Republic of Korea; 5 Department of Physiology, College of Medicine, Dong-A University, Busan, Republic of Korea; 6 Department of Laboratory Medicine, Jeonbuk National University Medical School and Hospital, Jeonju, Republic of Korea; 7 Department of Laboratory Medicine, Chonnam National University Medical School and Chonnam National University Hwasun Hospital, Hwasun, Republic of Korea; The Ohio State University, UNITED STATES

## Abstract

Phosphate-buffered saline (PBS) and Alsever’s solution (AS) are frequently used as media in blood-related studies, while 0.9% normal saline (NS) is frequently used in transfusion medicine. Despite the frequent use, the effects of these solutions on the shape and volume of red blood cells (RBCs) have not been reported. We collected blood samples from five healthy adults and used three-dimensional refractive index tomography to investigate the changes in the morphology of RBCs caused by changes in osmolality and solutes at the single-cell level. After diluting 2 μL of RBCs 200-fold with each solution (PBS, AS, and 0.9% NS), 40 randomly selected RBCs were microscopically observed. RBC shape was measured considering sphericity, which is a dimensionless quantity ranging from 0 (flat) to 1 (spherical). RBCs in plasma or AS showed a biconcave shape with a small sphericity, whereas those in 0.9% NS or PBS showed a spherical shape with a large sphericity. Moreover, we confirmed that sodium chloride alone could not elicit the biconcave shape of RBCs, which could be maintained only in the presence of an osmotic pressure-maintaining substance, such as glucose or mannitol. Although 0.9% NS solution is one of the most commonly used fluids in hematology and transfusion medicine, RBCs in 0.9% NS or PBS are not biconcave. Therefore, as the debate on the use of NS continues, future clinical studies or applications should consider the effect of glucose or mannitol on the shape of RBCs.

## Introduction

Red blood cells (RBCs) are highly differentiated cells, lacking all cell organelles, including the nucleus. Normal RBCs have been shown to exhibit an axially symmetric biconcave disc shape, typically with a diameter of approximately 7.8 μm and a thickness of approximately 2.5 μm [1]. The cytoplasm, the content of the RBCs, which is surrounded by the membrane, has a volume of approximately 94 μm^3^ at 300 mOsm/kgH_2_O [2]. Several studies have described the changes in the shape and volume of RBCs due to various chemical agents and environmental conditions [1, 3–5]. Particularly, the shape and volume of RBCs have been reported to be highly affected by osmolality and solutes, and in clinical practice, a biocompatible solution, such as 0.9% sodium chloride solution, which is considered to be isotonic, is frequently used with blood [6]. Previous studies have been conducted using changes in concentration of sodium chloride solution. It has been reported that the higher the concentration, the lower the RBC volume, and that hemolysis is induced when concentration is above 5.85% [7]. Moreover, the increase in osmotic pressure can change the biophysical properties of RBCs including radius, surface area, volume, viscosity, deformability [8], and hemoglobin concentration [9].

Both phosphate-buffered saline (PBS) and Alsever’s solution (AS) are frequently used as media in blood-related studies [10, 11]. Particularly, PBS, which is composed of 0.9% sodium chloride, 0.0795% sodium phosphate dibasic, and 0.0144% potassium phosphate monobasic, is a buffer solution commonly used in biological research. The composition of PBS is known to match the osmotic pressure and ion concentration of the human body. Similarly, AS is an isotonic solution suggested by Alsever in 1941, consisting of 0.42% sodium chloride, 2.05% glucose, 0.8% trisodium citrate, and 0.055% citric acid [12]. This solution is primarily used for storing blood and RBCs [13, 14]. Although PBS and AS are frequently used in blood-related experiments, the shape and volume of RBCs in these solutions have not been reported.

In general, an automated blood cell counter is necessary to analyze hematological parameters of RBCs, including the cell volume [15]. However, as this equipment is used to measure and calculate the hemotological parameters using impedance or light scattering methods after dilution of the blood sample, it is difficult to investigate the morphology of RBCs directly and examine the changes in the shape and volume of RBCs caused by changes in osmolality [16, 17]. In addition, cell surface area and sphericity have been addressed using indirect methods like osmotic feasibility tests or flow-based imaging methods with low precision and technical limitations [18, 19]. Recently, a three-dimensional (3D) quantitative phase-imaging technique has been widely used for analyzing RBCs at the single-cell level [20, 21]. This technique has the potential to be applied for improved understanding of RBCs and for characterizing the hematological parameters of individual RBCs [9, 22]. Therefore, this study aimed to investigate the changes in the morphology of RBCs caused by changes in osmolality and the concentration of solutes using engineering techniques.

## Materials and methods

### Study design and preparation of solutions

This study was conducted to observe the shape of RBCs according to the osmolality and concentration of solutes, and all experiments were performed using blood samples collected from five healthy adults. Blood was collected in an EDTA tube, and after centrifugation at 2500 rpm for 5 min, the plasma was separated without disturbing the buffy coat. RBCs were then divided into three groups and added to plasma, PBS (Thermo Fisher Scientific, Waltham, MA, USA), or AS (Sigma-Aldrich, St. Louis, MO, USA). For conducting additional experiments, we used 0.9% and 0.45% sodium chloride solutions (CJ Healthcare, Seoul, Korea), as well as 3% sodium chloride solution, glucose, and mannitol solutions (Daihan Pharmaceutical, Seoul, Korea), which are used as clinical solutions. Because sodium and glucose are the most differing factors in the PBS and AS, and are the main factors that determine the effective osmolality of serum [23], we performed the experiments of mixing sodium with glucose or mannitol to evaluate the effect of osmolality and solutes on the morphology of RBCs. The level of sodium chloride was fixed at 0%, 0.15%, 0.3%, 0.45%, 0.6%, or 0.75%, and glucose or mannitol was added to the each sodium chloride solution to increase the respective osmolality, including the normal reference range of serum osmolality (275–295 mOsm/KgH_2_O) [23]. The osmolality of the mixed solution was measured using the Fiske 2020 osmometer (Advanced Instruments, Norwood, MA, USA).

### Analysis of biochemical properties of blood

Before performing the experiments, the blood samples of all subjects were tested. Venous blood was collected after the subjects fasted for at least 8 h to ensure the quality of the samples and minimize the total preanalytical variability [24]. Subsequently, the blood samples were transferred to an EDTA tube and refrigerated at 4°C before performing the tests and experiments. All blood tests and experiments were performed within 6 hours after blood collection. A complete blood count test was conducted using the Sysmex XN1000 automated blood cell counter (Sysmex, Kobe, Japan) [16]. Blood urea nitrogen, creatinine, total protein, albumin, sodium, potassium, and chloride levels were measured using the Cobas 8000 c702 modular analyzer (Roche, Penzberg, Germany).

### Determination of morphological parameters of RBCs

The morphology of individual RBCs was determined by a common-path diffraction optical tomography (cDOT) setup microscope (HT-1H, Tomocube, Daejeon, Korea) using a 3D quantitative phase-imaging technique. This method helps reconstruct the 2D or 3D tomography of individual cells without labelling based on the common-path laser interferometry and optical diffraction tomography principles. To ensure RBCs were unaffected by the surrounding cells, 2 μL of collected blood was diluted by a factor of 200 with each solution. The diluted samples were then loaded in Tomodish (Tomocube), which is a specialized cell dish providing proper conditions for obtaining holotomographic images. Subsequently, 40 randomly selected RBCs were observed under the microscope [25, 26]. The RBCs were observed within 30 minutes after exposure to a new solution. Commercial software (Tomostudio, Tomocube) was used to visualize and analyze the measured 3D refractive index using phase-retrieval algorithm to retrieve the amplitude and phase images of the RBCs. Data on the morphological parameters of RBCs were obtained using refractive index threshold values and implicit function (regionprops3) in MATLAB (Mathwork, Natick, MA, USA). Further details of the equipment and software can be found in previous studies [27, 28]. For quantitative comparison of the morphology of RBCs in the different solutions, we calculated two morphological parameters, namely the aspect ratio and sphericity, which are described in previous papers for measuring the morphology of RBCs including discocytes, echinocytes, and spherocytes [26, 29]. The aspect ratio, a dimensionless quantity ranging from 0 to 1, was calculated as the ratio of diameters of the long to short axes of each RBC. The long and short axes were determined by fitting an ellipse to the cell boundary [30]. The sphericity, a dimensionless quantity ranging from 0 to 1, was obtained by the following equation: sphericity = π^1/3^(6V)^2/3^/A, where V represents the volume and A represents the surface area. Based on this parameter, a sphericity of 1 indicates a spherical shape, whereas 0 indicates a flat shape [31].

### Statistical analyses

When comparing the morphological parameters of RBCs between the three groups (plasma, PBS, and AS), ANOVA was performed followed by Bonferroni’s post-hoc analysis. When the sphericity was evaluated according to the solution, the *t*-test was applied for independent comparisons. All statistical analyses were performed using the GraphPad Prism 8 software (GraphPad, San Diego, CA, USA) and R version 3.6.0 (https://www.r-project.org). Statistical significance was set at p < 0.05.

### Ethical consideration

The study protocol was approved by the Institutional Review Board of the Gwangju Institute of Science and Technology (20200302-BR-52-01-01). All participants provided informed consent. This study conforms to the principles outlined in the Declaration of Helsinki, 7^th^ revision of 2013.

## Results

### Biochemical characteristics of blood

All subjects showed normal hematology and blood chemistry values in laboratory tests. The average mean cell volume of RBCs was estimated to be 89.5 fL. The mean concentration of sodium and glucose, and mean osmolality were 140 mmol/L, 91 mg/dL, and 294 mOsm/kgH_2_O, respectively.

### Morphology of RBCs in the conventional solutions

**Fig 1:**
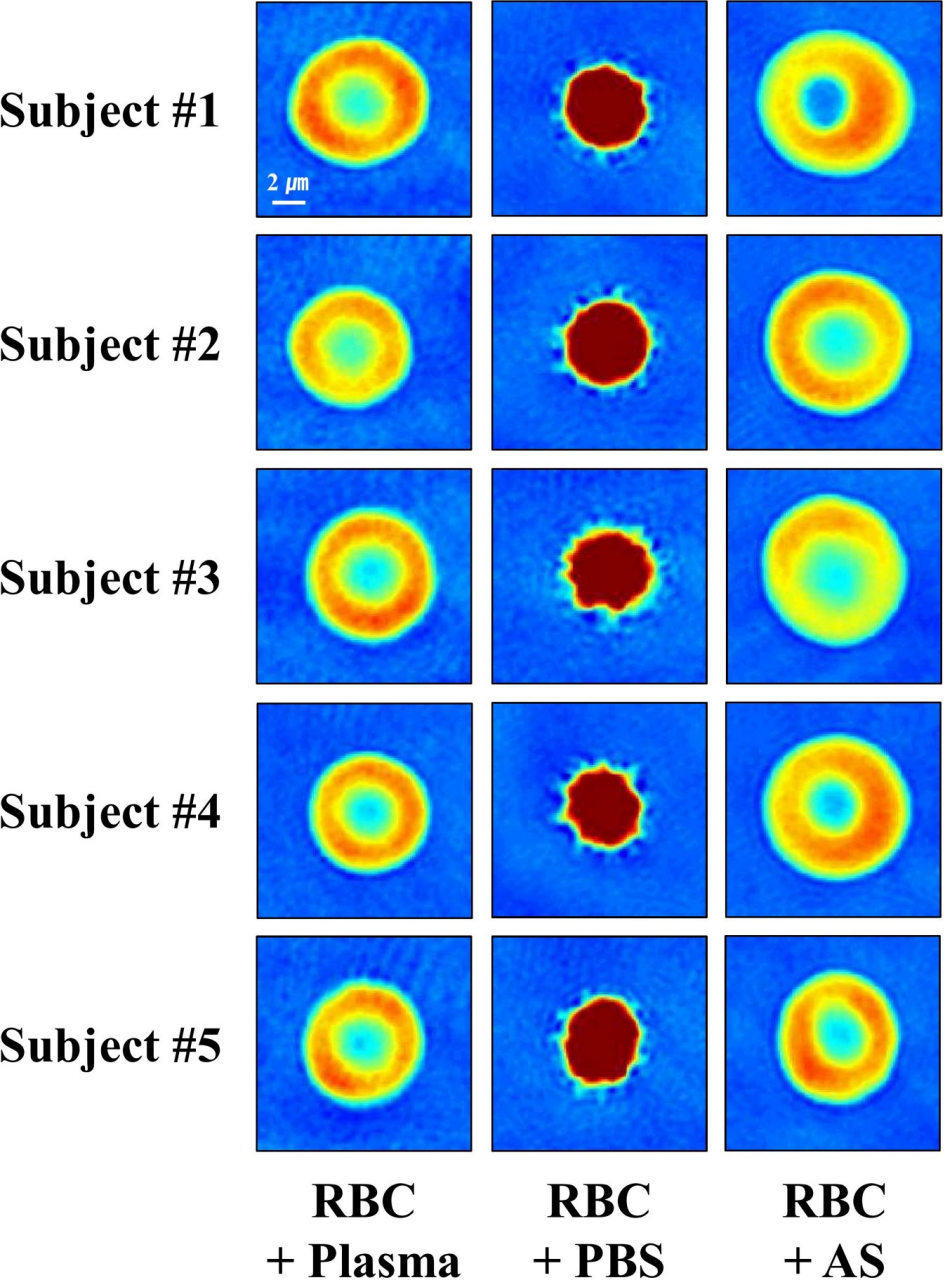
2D images presenting the morphology of RBCs in the conventional solutions. RBC, red blood cell; PBS, phosphate-buffered saline; AS, Alsever’s solution.

To investigate the morphology of RBCs in the solutions, individual RBCs were observed using cDOT. As shown in Fig 1, RBCs in plasma or AS displayed a biconcave shape, whereas those in PBS showed a spherical shape. This was consistent across the tested samples obtained from all subjects. Additionally, 3D images of RBCs in the three solutions are shown in S1 Fig. Similar to the observations from the 2D images, RBCs in plasma or AS showed a biconcave disc shape, whereas RBCs in PBS exhibited a spherical shape. Furthermore, although the volumes of individual RBCs in 1.05%, 0.9%, 0.75%, 0.6%, and 0.45% sodium chloride solutions varied, their shapes remained spherical (S2 Fig).

### Comparison of the morphological parameters of RBCs in the conventional solutions

**Fig 2:**
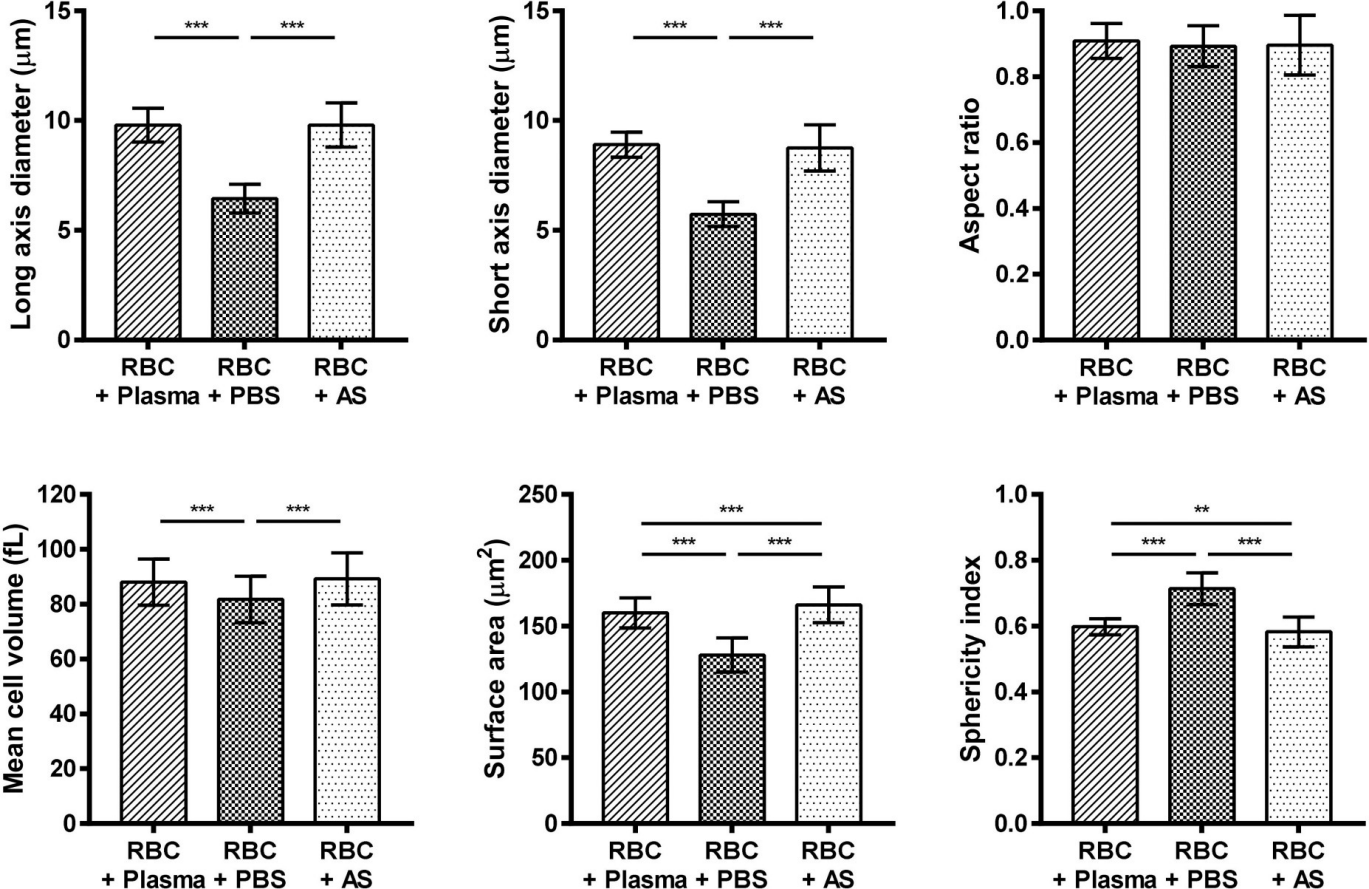
Comparison of the morphological parameters of RBCs in the conventional solutions. Asterisks correspond to the following p-values: * p < 0.05, ** p < 0.01, *** p < 0.001. RBC, red blood cell; PBS, phosphate-buffered saline; AS, Alsever’s solution.

After analysis of the morphological characteristics of RBCs, the parametric data obtained were compared according to the solution used, as shown in Fig 2. We observed that the diameters of the long and short axes were significantly smaller in RBCs in PBS (long axis: 6.44 ± 0.65 μm; short axis: 5.74 ± 0.57 μm; p < 0.001) compared with that in RBCs in plasma or AS. However, significant differences in the aspect ratio were not observed among the three groups. In the case of the mean cell volume, the values were 88.06 ± 8.43 fL and 89.27 ± 9.48 fL for RBCs in plasma and AS, respectively; no significant differences were observed between the two groups. However, RBCs in PBS were found to exhibit a significantly smaller mean cell volume of 81.77 ± 8.51 fL (p < 0.001) compared with RBCs in plasma or AS. We also found that the surface area of RBCs was the smallest in PBS (128.11 ± 12.94 μm^2^), whereas it was the highest in AS (166.10 ± 13.57 μm^2^). The sphericity of RBCs was estimated to be the highest in PBS (0.71 ± 0.05), whereas it was the lowest in AS (0.58 ± 0.05). Conclusively, we observed that both the surface area and sphericity significantly differed among the three groups (p < 0.01).

### Morphology of RBCs in sodium chloride and glucose solutions

**Fig 3:**
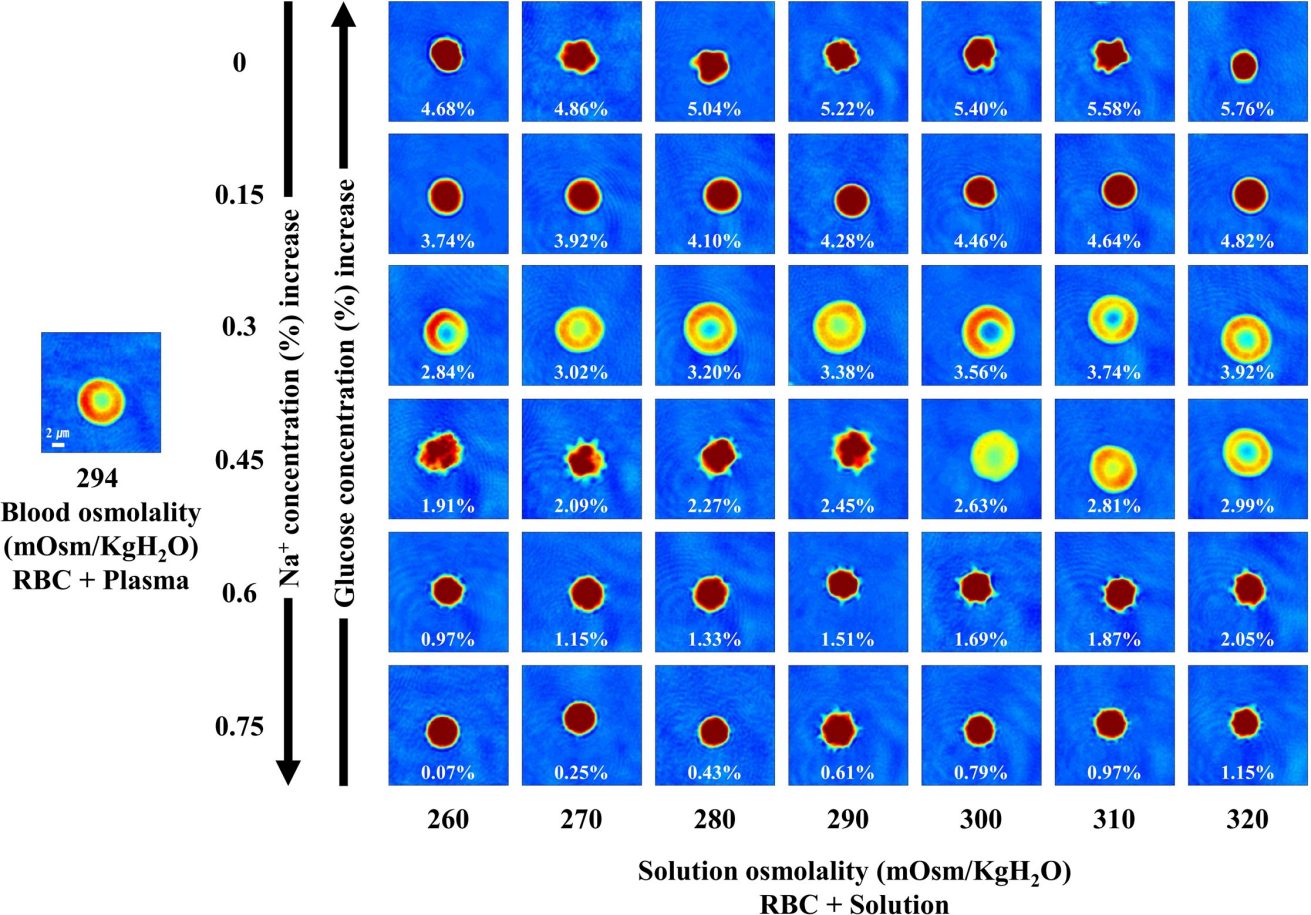
2D images presenting the morphology of RBCs for one healthy subject in sodium chloride and glucose solutions. The level of sodium chloride was fixed at 0%, 0.15%, 0.3%, 0.45%, 0.6%, and 0.75%, and glucose was mixed according to the respective osmolality. The value in each panel represents the concentration of glucose.

To analyze the effect of sodium and glucose on the shape of RBCs, we quantitatively investigated the shape of RBCs under different concentrations of sodium chloride and glucose at various osmolality levels. Fig 3 illustrates the changes in the biconcave shape of a RBC for one healthy subject under the above-mentioned conditions. As shown in S3 Fig, we did not observe any significant difference in the sphericity of RBCs between blood and solutions with 300–320 mOsm/kgH_2_O osmolality, 0.3% sodium chloride concentration, and 3.56–3.92 g/dL glucose concentration. To visualize the distribution of the sphericity according to the concentrations of sodium chloride and glucose, related-graphs were generated (S4 Fig). The dark, sky blue colored-data points indicate the aforementioned specific region highlighting concentrations of solutes resulting in the biconcave shape. The point indicating the sphericity of RBCs in AS was close to this specific region.

### Morphology of RBCs in sodium chloride and mannitol solutions

**Fig 4:**
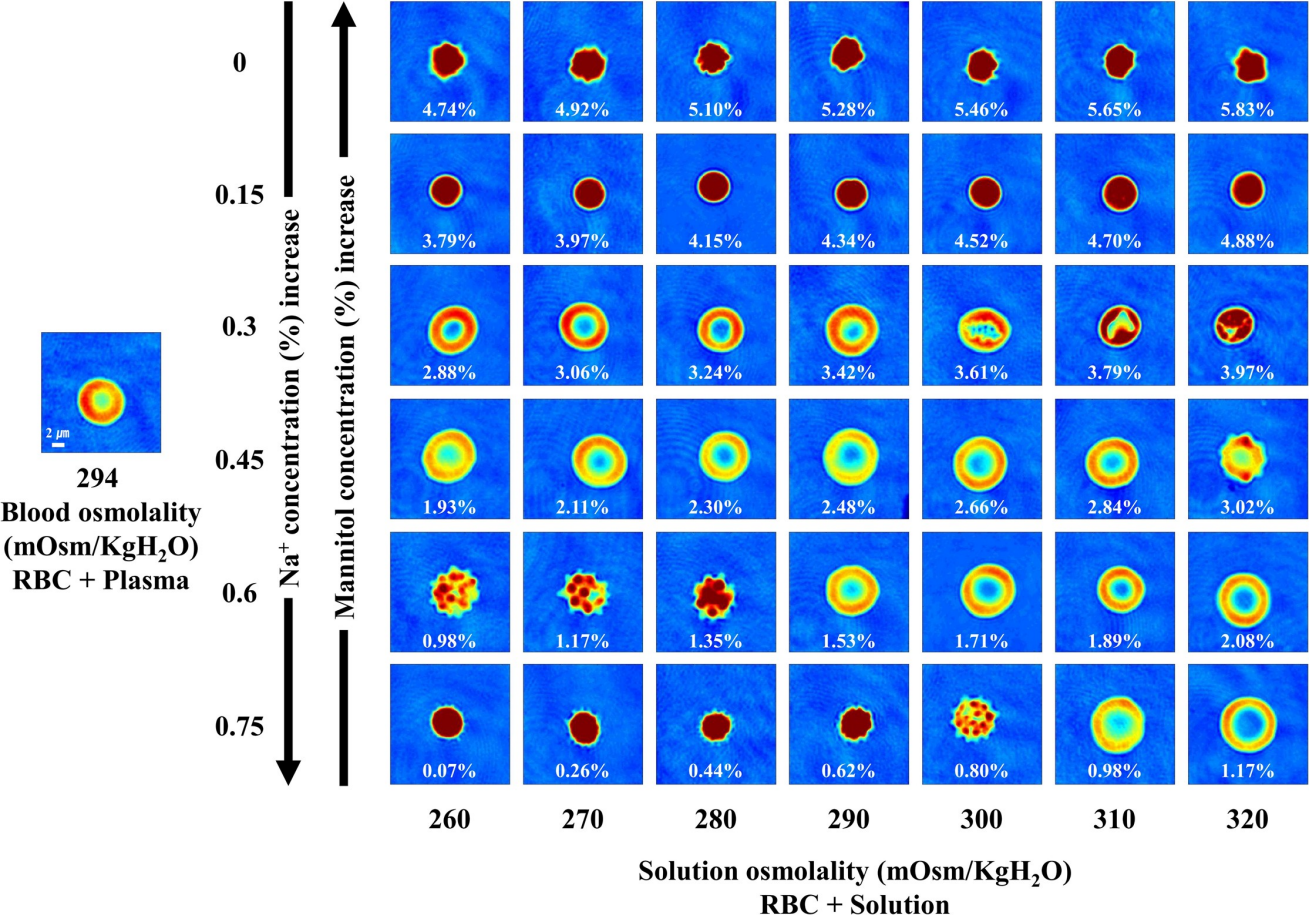
2D images presenting the morphology of RBCs for one healthy subject in sodium chloride and mannitol solutions. The level of sodium chloride was fixed at 0%, 0.15%, 0.3%, 0.45%, 0.6%, and 0.75%, and mannitol was mixed according to the respective osmolality. The value in each panel represents the concentration of mannitol.

To analyze the effect of solutes other than glucose on the shape of RBCs, we quantitatively investigated the shape of RBCs under different concentrations of sodium chloride and mannitol at various osmolality levels. Fig 4 illustrates the changes in the biconcave shape of a RBC for one healthy subject under the above-mentioned conditions. We found that the range of concentrations that helped retain the biconcave shape of RBCs was greater for the sodium chloride and mannitol solution than that observed in the case of sodium chloride and glucose solution. As shown in S5 Fig, we did not detect any significant difference in the sphericity of RBCs between blood and solutions with an osmolality of 260 or 270 mOsm/kgH_2_O, a sodium chloride concentration of 0.3%, and a mannitol concentration of 2.88 or 3.06%. Although we did observe the biconcave shape of RBCs in solutions with sodium chloride concentrations ranging from 0.45% to 0.75%, their sphericities were significantly lower than that of RBCs in plasma. The sphericity distribution according to the concentrations of sodium chloride and mannitol is illustrated using related-graphs (S6 Fig).

## Discussion

RBCs are known to change shapes under various external conditions, especially with changes in the sodium chloride concentration and osmolality [1]. A 0.9% sodium chloride solution is considered isotonic and is one of the most commonly used fluids in hematology and transfusion medicine. This solution is also used for performing intravenous infusion with blood, conducting washing steps, salvaging RBCs, or subjecting platelets to washing procedures. However, recent studies have reported that NS does not match the human physiological conditions and may be toxic [32, 33]. Kirkley et al. reported that NS causes greater hemolysis during conduction of washing steps and short-term storage (24 h or less) of RBCs compared with other solutions [34]. Masalunga et al. reported that subjecting erythrocytes to washing steps with saline causes increased hemolysis in neonatal extracorporeal membrane oxygenation recipients compared with those receiving unwashed erythrocytes [35]. Additionally, Yang et al. reported that conduction of intraoperative salvage with pre-infusion washing, using a buffered solution containing mannitol, adenine, and phosphate, reduces the dysfunction of RBCs and hemolysis compared with NS [36]. Although PBS, which is similar to 0.9% sodium chloride solution, and AS, which has a composition similar to 0.45% sodium chloride solution, have been used in several biological studies, to the best of our knowledge, there are currently no reports discussing the morphology of RBCs in these solutions. Owing to the current scenario of increasing concerns regarding the use of NS, this study aimed to investigate the morphology of RBCs in PBS and AS.

Morphological analysis of RBCs using cDOT revealed that RBCs in AS showed a biconcave disc shape similar to those of RBCs in plasma. When comparing the morphological parameters of RBCs in different solutions, we observed that both the mean cell size and volume were relatively low for RBCs in PBS, and were not significantly different between RBCs in plasma and AS. The surface area of RBCs was demonstrated to be the highest in AS, whereas it was the lowest in PBS. Consequently, we detected that the sphericity of RBCs in PBS was close to 1, indicating a relatively spherical shape. The surface area and sphericity of RBCs in AS were also shown to be close to the values observed for RBCs in plasma, but significant differences were observed between RBCs in plasma and AS. This finding may be attributed to the presence of various proteins, mainly albumin, contributing to the membrane stabilization, which may affect the shape and volume of RBCs [37, 38].

After confirming that RBCs exhibited a spherical shape in PBS and solutions of various concentrations of sodium chloride, we further investigated the shape of RBCs under conditions of different concentrations of sodium and glucose at various osmolalities. We accordingly found that the shape of RBCs was biconcave in solutions with 0.3–0.45% sodium chloride and 2.63–3.92 g/dL glucose. Citrate-phosphate-dextrose (CPD) and CPD solution with adenine (CPD-A), which are used as anticoagulant preservative solutions, contain 2.55 g/dL and 3.19 g/dL of glucose, respectively, close to the concentration of glucose in RBCs showing a biconcave shape, as illustrated in Fig 3. In 1915, Rous and Turner reported that glucose decelerates the hemolysis of RBCs in saline, and by adding citrate, it is possible to store blood for up to 4 weeks with minimal haemolysis [39]. Anticoagulant preservative solutions, such as CPD and CPD-A, are developed based on this observation. In general, substances such as glucose and adenine are added to the storage solution for ATP production [40]. Similar to CPD and CPD-A, AS contains glucose, unlike PBS; thus, it may increase the storage period of blood. Disbro et al. reported that hemolysis is less frequent and stable when the blood of patients using daratumumab is stored in AS [11]. Furthermore, based on the results of the present study, it may be inferred that glucose plays an important role in maintaining the biconcave shape of RBCs, and acts as a substrate for ATP production. In fact, glucose is an essential factor in determining osmolality, along with sodium chloride [41]. Viskupicova et al. reported that hemolysis, eryptosis, and calcium accumulation are decreased when erythrocytes are exposed to glucose [42].

Additionally, we confirmed that mannitol, which can also induce changes in the osmotic pressure, led to the formation of a biconcave shape in RBCs. A number of additive solutions have been developed to extend the storage period of RBCs up to 6 weeks [43], after separation of blood into RBCs and plasma in clinical settings. Importantly, most of these additive solutions contain mannitol for the prevention of hemolysis and protection of the cell membrane [40].

Nonetheless, this study had some limitations. First, we did not analyze the effect of osmolality on the shape of RBCs under the various conditions tested because of the small sample size, and experiments were performed only on healthy subjects. In addition, we investigated the limited number of RBCs comparing to that usually investigated using an automated blood analyzer. Second, additional research is warranted on the effects of adenine and phosphate on the shape of RBCs. Lastly, few studies have reported that when RBCs are continuously exposed to glucose, lipid peroxidation increases, whereas the enzymatic activity of erythrocytes decreases [42, 44]. Therefore, further research is necessary to determine whether glucose is beneficial or harmful during the storage of RBCs.

In conclusion, this study confirmed that the presence of sodium chloride alone could not elicit the biconcave shape of RBCs, and the biconcave shape could be maintained only in the presence of an osmotic pressure-maintaining substance, such as glucose or mannitol. While the debate on the use of NS with blood continues, future clinical studies will be necessary to evaluate the effect of glucose and mannitol on the shape of RBCs.

## Supporting information

S1 Fig Reconstructed 3D images of red blood cells in the conventional solutions. RBC, red blood cell; PBS, phosphate-buffered saline; AS, Alsever’s solution.
(DOCX)

S2 Fig 2D images of red blood cells in the sodium chloride solutions. RBC, red blood cell.
(DOCX)

S3 Fig Comparison of sphericity according to the solutions (Sodium chloride & Glucose). The p-value was obtained from the Student’s t-test performed for sphericity of red blood cells in plasma (reference, on the left) and in the respective solution. Asterisks correspond to the following p-values: * p-value < 0.05, ** p-value < 0.01, *** p-value < 0.001. RBC, red blood cell.
(DOCX)

S4 Fig Sphericity according to the solutions (Sodium chloride & Glucose). PBS, phosphate-buffered saline; AS, Alsever’s solution.
(DOCX)

S5 Fig Comparison of sphericity according to the solutions (Sodium chloride & Mannitol). The p-value was obtained from the Student’s *t*-test performed for sphericity of red blood cells in plasma (reference, on the left) and in the respective solution. Asterisks correspond to the following p-values: * p-value < 0.05, ** p-value < 0.01, *** p-value < 0.001. RBC, red blood cell.
(DOCX)

S6 Fig Sphericity according to the solutions (Sodium chloride & Mannitol). (DOCX)
