# Supplementary figures and images for: Effects of osmolality and solutes on the morphology of red blood cells according to three-dimensional refractive index tomography
Source: PLoS One. 2021 Dec 31;16(12):e0262106. doi: 10.1371/journal.pone.0262106 (PMC8719701; doi:10.1371/journal.pone.0262106)

**
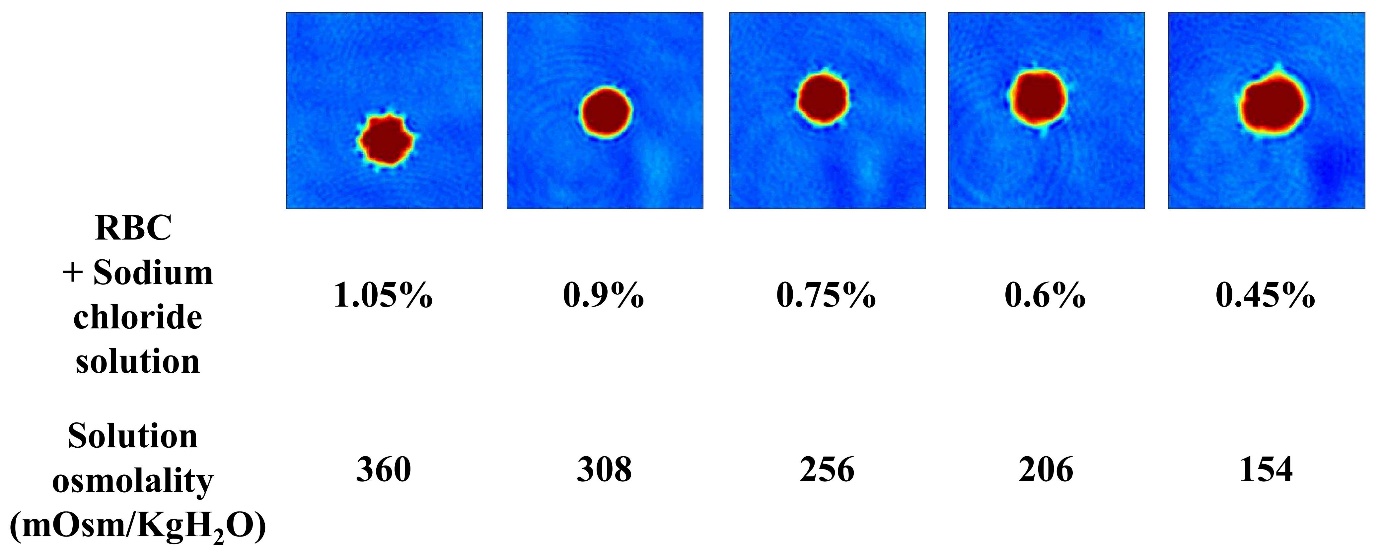
**

**S2 Fig. 2D images of red blood cells in the sodium chloride solutions.** RBC, red blood cell.

Supplement: S2 Fig — RBC, red blood cell. (DOCX) [file pone.0262106.s002.docx]

**
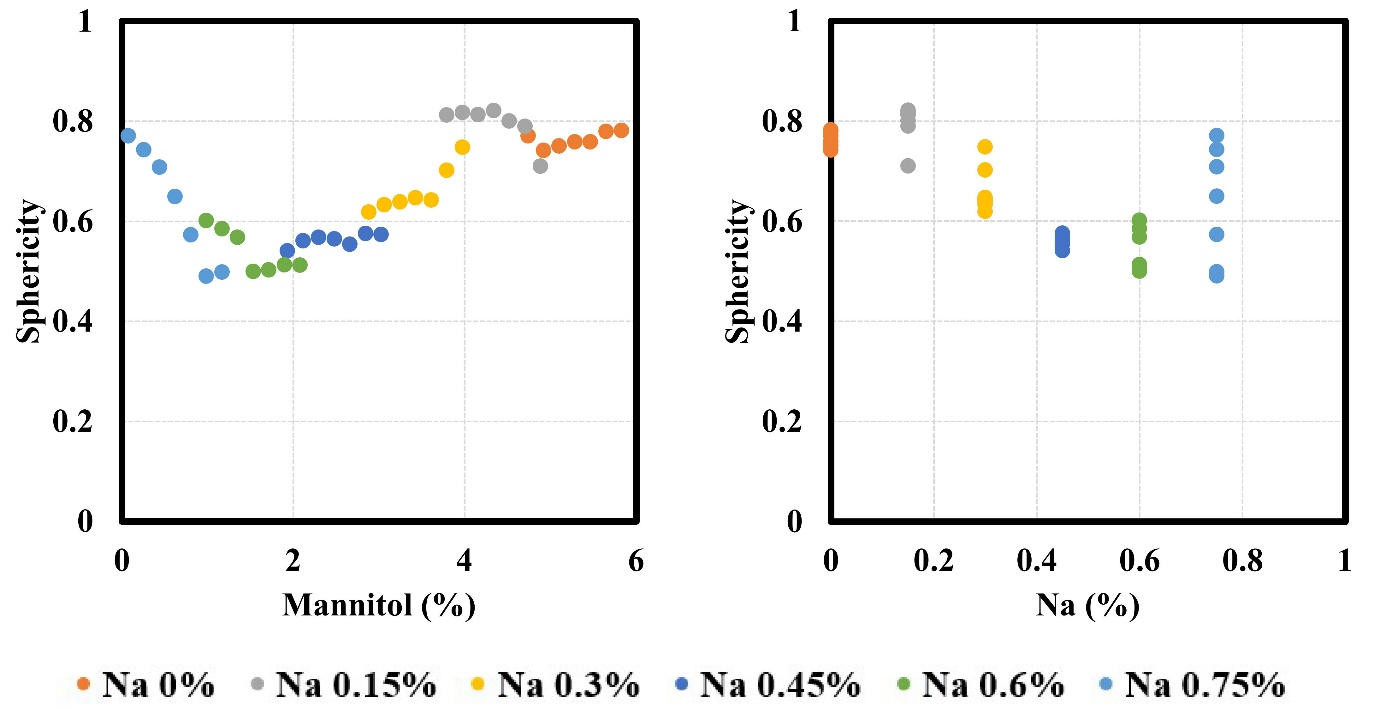
**

**S6 Fig. Sphericity according to the solutions (Sodium chloride & Mannitol).**

Supplement: S6 Fig — (DOCX) [file pone.0262106.s006.docx]
