# Supplementary material for: Effects of osmolality and solutes on the morphology of red blood cells according to three-dimensional refractive index tomography
Source: PLoS One. 2021 Dec 31;16(12):e0262106. doi: 10.1371/journal.pone.0262106 (PMC8719701; doi:10.1371/journal.pone.0262106)
Supplement: S3 Fig — The p-value was obtained from the Student’s t-test performed for sphericity of red blood cells in plasma (reference, on the left) and in the respective solution. Asterisks correspond to the following p-values: * p-value < 0.05, ** p-value < 0.01, *** p-value < 0.001. RBC, red blood cell. (DOCX) [file pone.0262106.s003.docx]

**
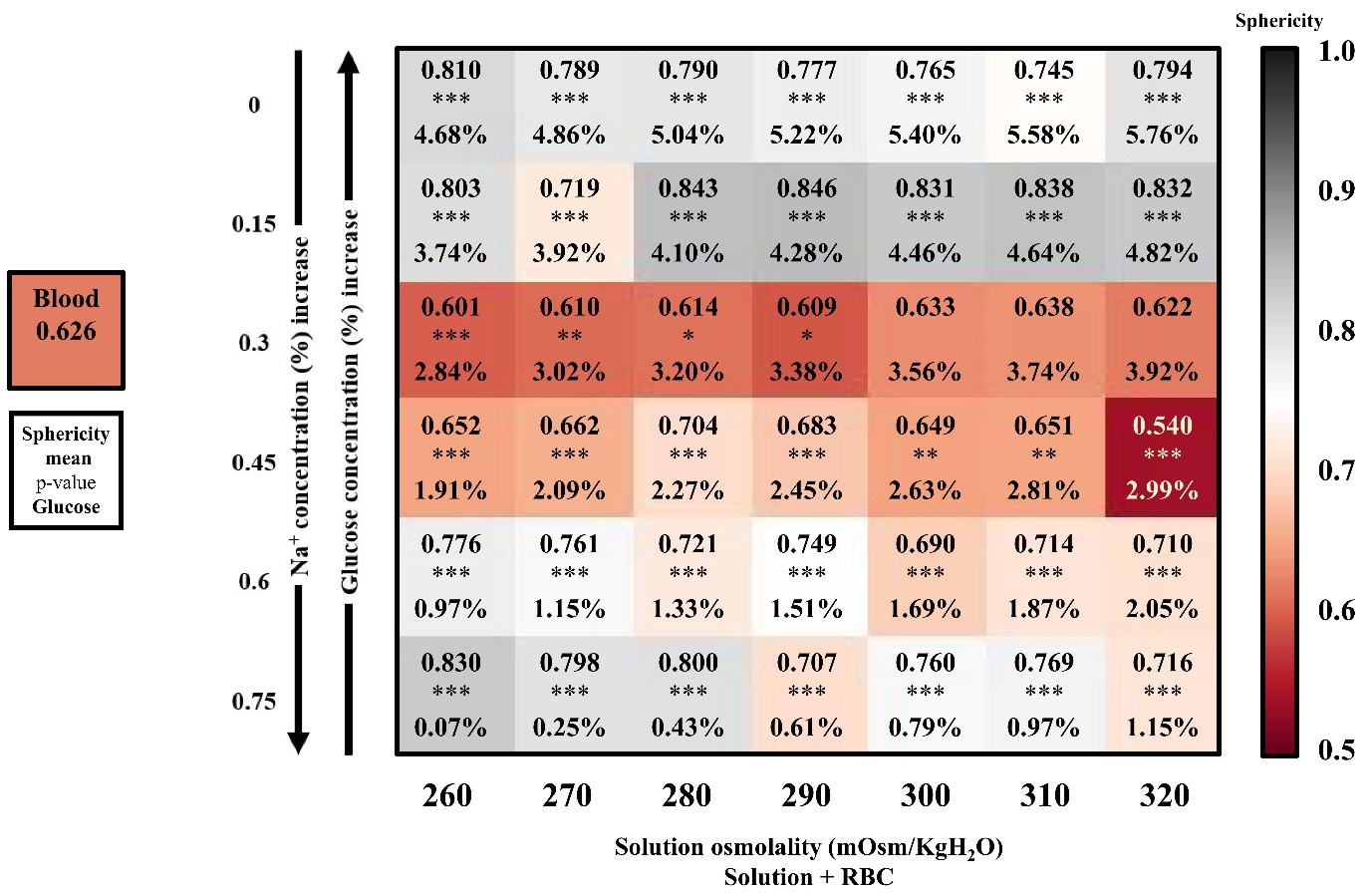
**

**S3 Fig. Comparison of sphericity according to the solutions (Sodium chloride & Glucose).** The p-value was obtained from the Student’s *t*-test performed for sphericity of red blood cells in plasma (reference, on the left) and in the respective solution. Asterisks correspond to the following p-values: * p-value < 0.05, ** p-value < 0.01, *** p-value < 0.001. RBC, red blood cell.
