# Supplementary material for: Effects of osmolality and solutes on the morphology of red blood cells according to three-dimensional refractive index tomography
Source: PLoS One. 2021 Dec 31;16(12):e0262106. doi: 10.1371/journal.pone.0262106 (PMC8719701; doi:10.1371/journal.pone.0262106)
Supplement: S4 Fig — PBS, phosphate-buffered saline; AS, Alsever’s solution. (DOCX) [file pone.0262106.s004.docx]

**
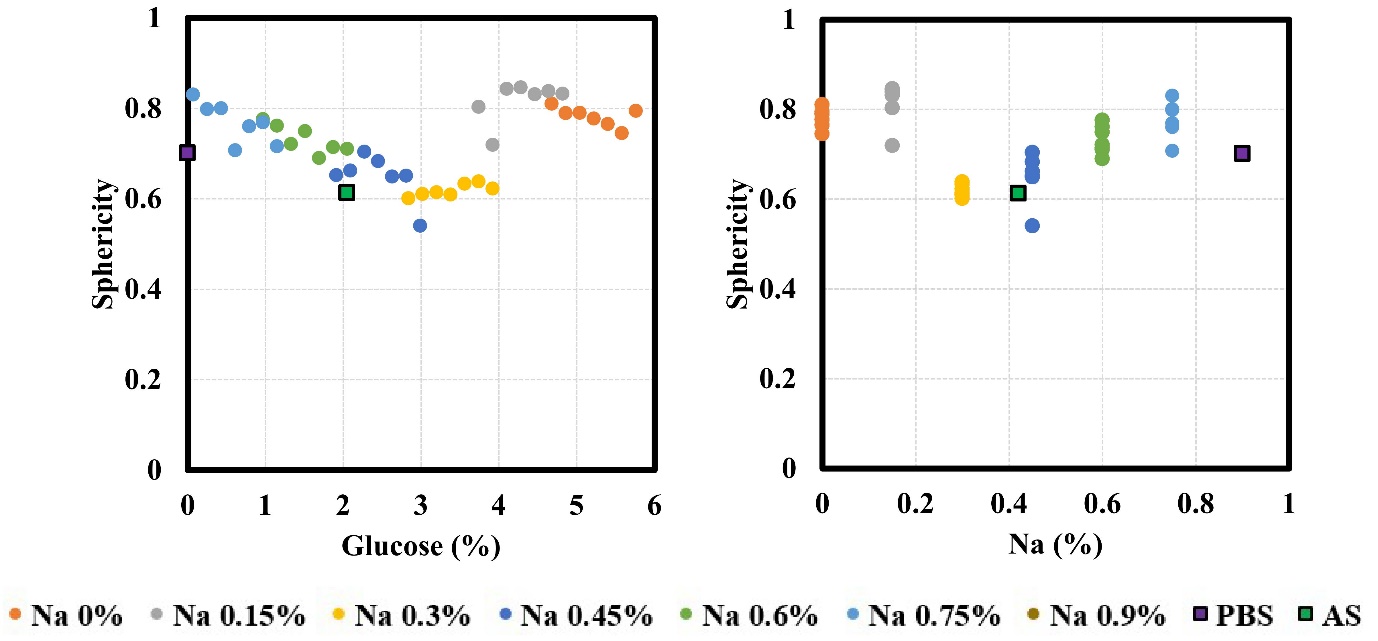
**

**S4 Fig. Sphericity index according to the solutions (Sodium chloride & Glucose).** PBS, phosphate-buffered saline; AS, Alsever’s solution.
